# Biodiversity of the beneficial soil-borne fungi steered by *Trichoderma*-amended biofertilizers stimulates plant production

**DOI:** 10.1038/s41522-023-00416-1

**Published:** 2023-07-05

**Authors:** Yan Wang, Zhengyang Liu, Xinyi Hao, Ziqi Wang, Zhe Wang, Shanshan Liu, Chengyuan Tao, Dongsheng Wang, Bei Wang, Zongzhuan Shen, Qirong Shen, Rong Li

**Affiliations:** 1 Jiangsu Provincial Key Lab of Solid Organic Waste Utilization, Jiangsu Collaborative Innovation Center of Solid Organic Wastes, Educational Ministry Engineering Center of Resource-saving fertilizers, Laboratory of Bio-interactions and Crop Health, Nanjing Agricultural University, Nanjing, 210095 Jiangsu P. R. China; 2 The Sanya Institute of Nanjing Agricultural University, Sanya, 572000 Hainan P. R. China; 3 Nanjing Institute of Vegetable Science, Nanjing, 210042 Jiangsu P. R. China

**Keywords:** Microbiome, Applied microbiology

## Abstract

The soil microbiota is critical to plant performance. Improving the ability of plant-associated soil probiotics is thus essential for establishing dependable and sustainable crop yields. Although fertilizer applications may provide an effective way of steering soil microbes, it is still unknown how the positive effects of soil-borne probiotics can be maximized and how their effects are mediated. This work aims to seek the ecological mechanisms involved in cabbage growth using bio-organic fertilizers. We conducted a long-term field experiment in which we amended soil with non-sterilized organic or sterilized organic fertilizer either containing *Trichoderma guizhouense* NJAU4742 or lacking this inoculum and tracked cabbage plant growth and the soil fungal community. *Trichoderma*-amended bio-organic fertilizers significantly increased cabbage plant biomass and this effect was attributed to changes in the resident fungal community composition, including an increase in the relative abundance and number of indigenous soil growth-promoting fungal taxa. We specifically highlight the fundamental role of the biodiversity and population density of these plant-beneficial fungal taxa in improving plant growth. Together, our results suggest that the beneficial effects of bio-organic fertilizer seem to be a combination of the biological inoculum within the organic amendment as well as the indirect promotion through effects on the diversity and composition of the soil resident plant-beneficial fungal microbiome.

## Introduction

Soils are essential to human health because they supply feed, food, fiber, and medicine^1,2^. Soil microorganisms play a key role in providing a variety of services necessary for plant growth^3^. Plants can manipulate the soil microbiome by supplying resources, thereby recruiting microorganisms to colonize their roots^4,5^, which in turn affects plant performance^6,7^. Root-associated microorganisms that are beneficial to host plants have been reported to be essential for providing the following ecosystem services^8^: organic matter decomposition, nutrient cycling, plant productivity, and pathogen control^9,10^. Despite the fact that many functions have been characterized, there are still many challenges to directionally enhance these plants advantageous functions stored in the soil microbiome. Consequently, a better understanding of deploying soil-native microorganisms to improve soil ecosystem function is essential for the development of sustainable agricultural systems.

Biodiversity has frequently been proven to be linked to ecosystem productivity and stability^11–13^. Microbial communities with high diversity have been reported to improve crop growth because they may occupy a wider range of ecological niches^14^ and maintain higher levels of enzyme activity^15^. However, some findings have also suggested that microbial community functioning, such as plant growth promotion and disease suppression, may be driven primarily by specific microbial groups^16,17^. These particular taxa increased nutrient availability, plant productivity, and disease resistance in natural ecosystems^16,18^. Furthermore, interspecific interactions within communities have also been reported to comprise resource competition, antagonistic action, and metabolic cross-feeding that can considerably determine microbial functionality^14,19,20^. Given the complexity of soil microbial interactions, the deployment of the soil microbiome to optimize plant-microbial partnerships is still an arduous assignment.

Recently, the introduction of exogenous microorganisms to the soil has been an effective strategy to increase microbiome-associated multifunctionality^21^. Inoculated microbes can directly enhance microbiome-associated functionality on the basis of their own functional properties, such as nitrogen fixation, nutrition mineralization, or pathogen control^22^. In addition to directly introducing functions into the soil, injected microbes could also induce indirect effects by stimulating the variety and composition of native microbiota and thereby affect the functioning of the resident soil microbiome^23–25^. Such alterations might be driven via microbial resources competition, antagonism triggered by antibiosis^26^, interactions with plants by altering exudation patterns in plant roots, and inducing systemic resistance in plants^27^. The introduction of plant probiotics by bio-organic fertilizer application could enhance plant health, nutrition, and stress tolerance^28–30^ by indirectly affecting the soil microbiome^31–33^; however, how and to what extent bio-organic fertilization stimulates and enhances the beneficial effects of plant-associated microbiomes in the field are still relatively poorly understood.

*Trichoderma* spp. are free-living opportunistic fungi that are common in soil and plant root systems and are widely used as a biological inoculum in agricultural production. Numerous studies have shown that root colonization by *Trichoderma* spp. could frequently enhance root growth and development, crop productivity, resistance to biotic/abiotic stresses, and the uptake and use of nutrients^34,35^. Although these plant-beneficial effects are mostly the result of direct effects on plants, the microbial ecological mechanisms explaining crop growth and yield promotion upon *Trichoderma* spp. inoculation in the field condition still remains largely unknown.

In this study, we established a Chinese cabbage-wild cabbage rotation field experiment for eight crop seasons, in which the soils were amended with sterilized or non-sterilized organic fertilizer inoculated with or without plant-beneficial fungal inoculant (*Trichoderma guizhouense* NJAU4742) and chemical fertilizer. This design allowed us to disentangle the relative contribution of the bio-organic fertilizer’s components comprised of organic substrate addition, the fertilizer microbiome, and the inoculated biocontrol strain on crop growth and yield. We tracked fungal communities using fungal ITS rRNA gene amplicon sequencing and culture-dependent experiments and sought to (i) determine the crop-growth promotion capability of *T*. *guizhouense* NJAU4742-amended bio-organic fertilizers and (ii) reveal the role of changes in the resident community and functioning of fungi after the introduction of *T*. *guizhouense* NJAU4742 in the crop-growth promotion. Our findings will eventually lay a foundation for deciphering the linkages between fertilization and soil microbiome manipulation aiming at promoting specific soil functions, such as crop yield promotion.

## Results

### Cabbage yield in the field experiment

**Fig. 1:**
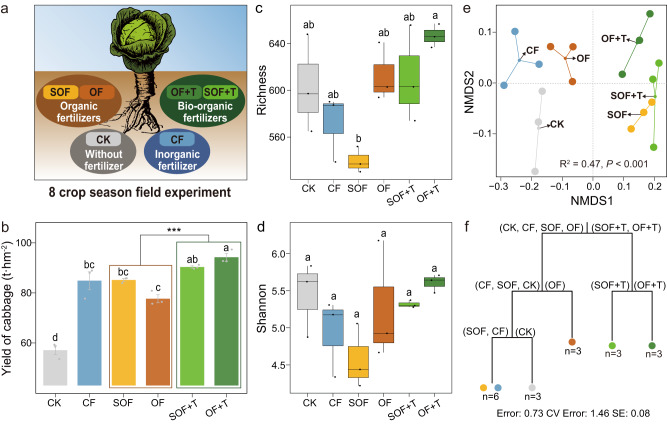
Cabbage yield and fungal community composition in the filed experiment. Long-term field experimental design (**a**). Cabbage yields of different treatments (mean ± SE) (**b**). Fungal richness (richness, **c**) and diversity (Shannon, **d**) indices of different treatments. Nonmetric multidimensional scaling (NMDS) ordinations of the fungal community based on the Bray–Curtis distance metric across all soil samples (**e**). Multiple regression tree (MRT) analysis of the treatment effects on the fungal community composition (**f**). The error, cross-validation error (CV error), and standard error (SE) of the MRT analysis are listed under the tree. CK control, CF chemical fertilizer treatment, SOF sterilized organic fertilizer treatment, OF: organic fertilizer treatment, SOF + T: *T. guizhouense* NJAU4742 inoculated sterilized organic fertilizer treatment; and OF + T: *T. guizhouense* NJAU4742 inoculated organic fertilizer treatment. Asterisks indicate significant differences between bio-organic fertilizer treatments (SOF + T and OF + T) and organic fertilizer treatments (SOF and OF) as determined by the *t* test (***P < 0.001; *n* = 6). Different letters indicate a significant difference at the 0.05 probability level according to Tukey’s test (*n* = 3). Box plot displays the first and third quartile, with the horizontal bar at the median and whiskers showing the most extreme data point, which is no >1.5 times the interquartile range from the box.

All fertilizer treatments significantly increased the yield of cabbage compared with the control treatment in field experiment (Tukey’s test, *P* < 0.05; Fig. 1b). Both bio-organic fertilizer treatments (SOF + T and OF + T) significantly increased the cabbage yield, with OF + T showing the highest yield compared to the organic fertilizer (OF and SOF) and chemical fertilizer (CF) treatments (Tukey’s test, *P* < 0.05; Fig. 1b). The cabbage yield in the OF treatment was substantially lower compared to the SOF + T and OF + T treatments (Tukey’s test, *P* < 0.05). Furthermore, significant higher cabbage yield was observed in the group of bio-organic fertilizer treatments (SOF + T and OF + T) compared to the group of organic fertilizer treatments (SOF and OF) (*t* test, *P* < 0.05).

### Fungal community composition

Fungal richness (richness) and diversity (Shannon) indices did not differ significantly among the CK, CF, OF, SOF + T, and OF + T treatments (Tukey’s test, *P* > 0.05), while significantly lower fungal richness index was observed in the SOF treatment than OF + T treatment (Tukey’s test, *P* < 0.05, Fig. 1c). SOF treatment also decreased the fungal richness and diversity indices compared to the OF, SOF + T, and OF + T treatments (Tukey’s test, *P* > 0.05, Fig. 1c, d). Furthermore, the richness and diversity indices in chemical fertilizer and sterile organic fertilizer treatments (CF and SOF) were lower than CK (Tukey’s test, *P* > 0.05, Fig. 1c, d).

Nonmetric multidimensional scaling (NMDS) analysis result clearly showed significant variations in fungal community composition across the different treatments (ADONIS, *R*^2^ = 0.47, *P* < 0.001, Fig. 1e). Overall, the fungal community composition in the OF + T, SOF + T and SOF treatments was rather similar and different from that in the OF, CF and CK treatments along the first axis (Fig. 1e). Multiple regression tree (MRT) analysis indicated that the main driver of fungal communities was *T*. *guizhouense* NJAU4742 application, followed by chemical and organic fertilizers application (Fig. 1f). The fungal communities could be divided into two major groups according to whether the soil had been amended with *T*. *guizhouense* NJAU4742, with a further separation in the organic fertilizer treatment (OF) and other treatments (CF, SOF, and CK) (Fig. 1f). In conclusion, the MRT analysis result showed that the fungal community compositions from the OF + T and SOF + T treatments were quite similar and significantly different from those of the other treatments (Fig. 1f).

### Responsive fungal taxa

**Fig. 2:**
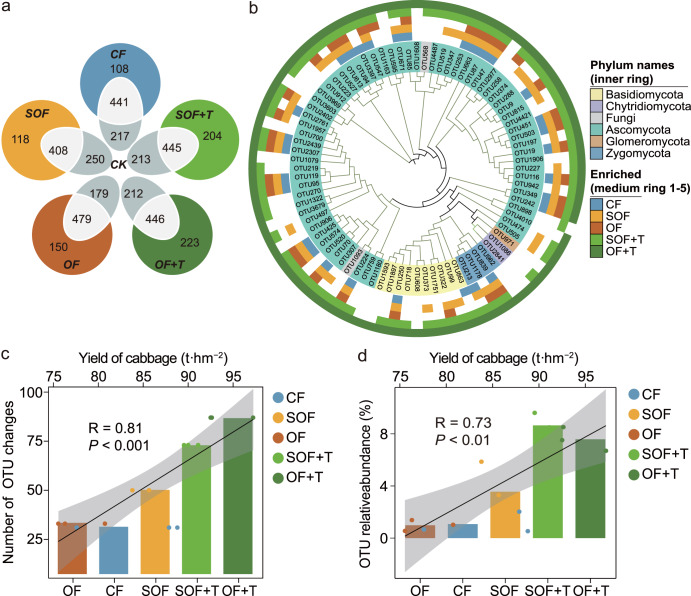
Responsive fungal taxa. Venn diagrams showing the number of fungal OTUs shared between CK with the CF, SOF, OF, SOF + T, and OF + T treatments or specific to each group (**a**). Cladogram showing phylogenetic relationships between 88 fungal OTUs (**b**). Leaf labels indicate representative sequence IDs. Rings, from the inner to the outside circles, represent (1) phylum-level taxonomy of OTUs; (2) shared and specific OTUs in CF treatment; (3) shared and specific OTUs in SOF treatment; (4) shared and specific OTUs in SOF + T treatment; (5) shared and specific OTUs in OF treatment; and (6) shared and specific OTUs in OF + T treatment. Bar graphs indicate the average number of potentially beneficial fungal OTUs in different treatments; linear regression relationship between the number of potentially beneficial fungal OTUs and cabbage yield (**c**). Bar graphs indicate the cumulative relative abundance of potentially beneficial fungal OTUs in different treatments; linear regression relationship between the cumulative relative abundance of potentially beneficial fungal OTUs and cabbage yield (**d**). CK control, CF chemical fertilizer treatment, SOF sterilized organic fertilizer treatment, OF organic fertilizer treatment, SOF + T *T. guizhouense* NJAU4742 inoculated sterilized organic fertilizer treatment; and OF + T: *T. guizhouense* NJAU4742 inoculated organic fertilizer treatment. Potentially beneficial fungal OTUs mean its relative abundance was significantly positively associated with yield.

To investigate the fungal OTUs that correlated with specific fertilization treatment, we used a Venn diagram to show the number of fungal OTUs shared between the CK treatment and different fertilizer treatments or specific to each treatment (Fig. 2a). Fungal OTUs in different fertilizer treatments were dominated by shared OTUs; in total, 80.32%, 77.57%, 76.15%, 68.57%, and 66.67% fungal species were shared between the CK treatment and the CF, SOF, OF, SOF + T, and OF + T treatments, respectively (Fig. 2a). Besides, Venn diagram result showed that 484 responsive fungal OTUs were shared between OF + T and SOF + T treatments, which was greater than the number of shared OTUs when compared SOF vs SOF + T, and OF vs OF + T, respectively (Supplementary Fig. 2a).

Among these responsive fungal taxa, Spearman’s rank correlation analysis found 88 fungal OTUs linked with cabbage yield (FDR < 0.05) and was defined as potentially beneficial fungi (Fig. 2b). Of these, 31, 50, 33, 73, and 87 potentially beneficial fungal OTUs were enriched in the CF, SOF, OF, SOF + T, and OF + T treatments, respectively. The largest number and highest cumulative relative abundances of potentially beneficial fungal OTUs were observed in the OF + T and SOF + T treatments compared with the CF, SOF, and OF treatments, respectively (Fig. 2c, d). Potentially beneficial fungal OTUs showed a particular distribution pattern in the soil treated with OF + T and SOF + T, prompting a more detailed examination. Linear regression results showed that the community richness (*P* < 0.001) and composition (*P* < 0.01) of potentially beneficial fungi, instead of overall soil fungal community richness (*P* > 0.05) and composition (*P* > 0.05), were significantly and positively correlated with cabbage yield (Supplementary Fig. 2b–e).

### Taxonomy of potentially beneficial fungi and their association with *T. guizhouense* NJAU4742

**Fig. 3:**
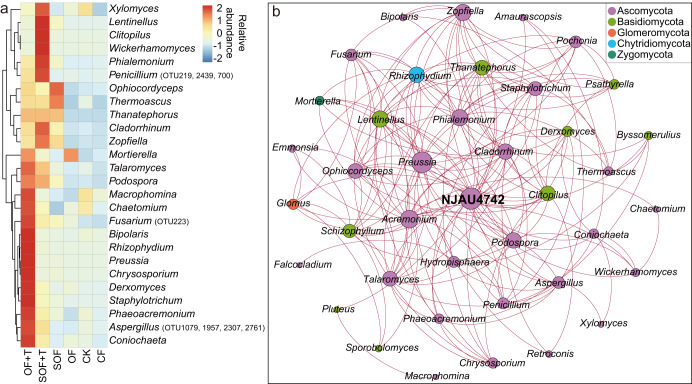
Taxonomy of potentially beneficial fungi and their association with *T*. *guizhouense* NJAU4742. Heatmap showing the average relative abundance of potentially beneficial fungi in different treatments at the genus level (**a**). Network analysis of potentially beneficial fungi and *T. guizhouense* NJAU4742 (**b**). Positive correlations are displayed in red. The nodes are colored according to different phyla. The size of each node is proportional to the betweenness centrality (Spearman’s |*r*|> 0.60, *P* value < 0.05).

We examined the classification of potentially beneficial fungal OTUs at the genus level and their relative abundances in different fertilizer treatments. Results showed that the potentially beneficial fungi were assigned to 39 genera, and most of them belonged to Ascomycota (Supplementary Fig. 3a). The potentially beneficial fungal genera were distributed in higher abundance in the two bio-organic fertilizer treatments (OF + T and SOF + T) compared to the other treatments, with OF + T showing the greatest variety and highest abundance (Fig. 3a). We further performed a network analysis of these potentially beneficial fungal genera and *T. guizhouense* NJAU4742 to identify their interrelationships. The network consisted of 40 nodes and 188 edges. The analysis showed that positive edges accounted for 100%, indicating that coexistence relationships occupied the entirety of this microbial network (Fig. 3b).

### Effects of fungal isolates on cabbage growth in the greenhouse experiment 1

In total, 59 and 39 fungal isolates were randomly collected from OF + T and OF soils, respectively, to confirm the findings of the fungal community analysis results (Supplementary Fig. 1). A total of 18 fungal genera could be distinguished among the 98 strains, including *Alternaria, Aureobasidium*, *Aspergillus*, *Cladosporium*, *Curvularia*, *Filobasidium*, *Epicoccum*, *Humicola*, *Irpex*, *Paecilomyces*, *Paracremonium*, *Purpureocillium*, *Sarocladium*, *Simplicillium*, *Stemphylium*, *Penicillium*, *Fusarium* and *Trichoderma*. In greenhouse experiment 1, twelve *Aspergillus* isolates, four *Penicillium* isolates, three *Fusarium* isolates, and ten *Trichoderma* isolates, which belonged to the potentially beneficial fungal groups, were selected to test their ability to promote the growth of cabbage. Pot trial results showed that most *Aspergillus*, *Penicillium,* and *Trichoderma* isolates could significantly promote the growth of cabbage (Supplementary Fig. 3b). On this basis, four fungal isolates (*Trichoderma* sp. t102, *Aspergillus* sp. t35, *Penicillium* sp. 45, and *Fusarium* sp. 53) with optimal growth promotion on cabbage were chosen for further study.

### Effects of co-inoculation of *T. guizhouense* NJAU4742 plus potentially beneficial fungal isolates in the greenhouse experiment 2

In greenhouse experiment 2, we further investigated the plant growth promotion efficiency of the fungal consortia comprised of *T. guizhouense* NJAU4742 and potentially beneficial fungi (*Trichoderma* sp. t102, *Aspergillus* sp. t35, *Penicillium* sp. 45, or *Fusarium* sp. 53). We noticed that the co-inoculation of *T. guizhouense* NJAU4742 together with the selected fungal isolates (*Trichoderma* sp. t102, *Aspergillus* sp. t35, *Penicillium* sp. 45, and *Fusarium* sp. 53) could significantly increase the cabbage biomass as compared to single fungal species (Supplementary Fig. 4a, b). In particular, the combination of *T. guizhouense* NJAU4742 plus *Penicillium* sp. 45 showed the strongest plant growth promotion capacity as compared to the other synthetic fungal consortia (Supplementary Fig. 4a, b).

### Effects of fungal community richness and population density on plant growth in the greenhouse experiments 3 and 4

**Fig. 4:**
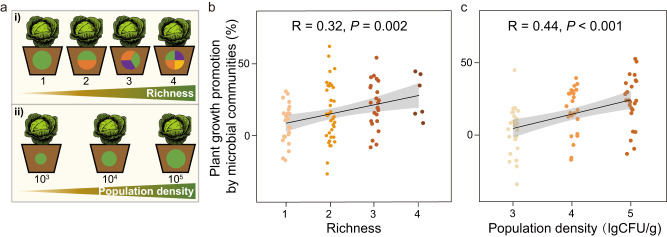
Effects of fungal community richness and population density on plant growth in the greenhouse experiments. Pot experimental design (**a**). Effects of four richness levels (1, 2, 3, and 4-strain communities) and three microbial population densities (10^3^, 10^4^, and 10^5^ CFU g^−1^ soil) on cabbage plant growth. Effects of fungal community richness on cabbage plant growth (**b**). Effects of fungal population density on cabbage plant growth (**c**). Plant growth promotion was calculated as the percentage increase in the fresh weight of treatments compared to the control.

In greenhouse experiments 3 and 4, we aimed to mechanistically understand the links between the richness and population densities of potentially beneficial fungi with plant growth (Fig. 4a). We found that increasing the richness and population densities of beneficial microbes improved the accumulation of plant biomass (Fig. 4b, c, Supplementary Tables 3 and 4). Nevertheless, some of the lower richness communities could express equally high levels of plant growth-promoting capacity as the 4-strain communities (Fig. 4b). Plant roots inoculated with the fungal community comprised of *Aspergillus* sp. t35, *Trichoderma* sp. t102, and *Fusarium* sp. 53 could increase the cabbage fresh weight by 36.49% ± 13.23% (mean ± SD), and this effect was stronger than that of the other combinations (Supplementary Table 3). In addition, random forest results showed that community richness and population densities were the key factors that mediated these positive effects (Supplementary Fig. 4c).

## Discussion

In this study, we investigated the impacts of bio-organic, organic, and chemical fertilization practices on the growth of cabbage within a crop rotation system in natural soil. Our objective was to explore the response of composition and functioning in soil-borne fungal microbiomes to different fertilization patterns and exogenous plant-beneficial fungi. We found that *Trichoderma*-amended bio-organic fertilizers (OF + T, SOF + T) application could form a more similar soil fungal community and stimulate a greater number of potentially beneficial soil-borne fungi that contribute to crop growth and yield. We further demonstrated that the soil-borne beneficial fungal diversity is significantly associated with crop yield using a biodiversity-ecosystem functioning approach. Together, these results highlighted the significance of the biodiversity of soil-borne beneficial phylotypes in sustaining crop output. This knowledge is important for providing the basis for the isolation and application of these microorganisms to increase crop production and support a constantly expanding global population.

Soil-borne microbiome is an important contributor to plant development, and the distinct shifts in soil microbial community diversity and composition have been closely linked to crop growth and yield^36,37^. We also found links between fertilization-induced changes in soil-borne fungal diversity and community composition with cabbage plant performance. The application of bio-organic, organic, and chemical fertilizers for eight cropping seasons significantly impacts the composition of soil-borne fungal community, which was associated with cabbage growth and yield. Previous studies have demonstrated that differences in microbial community composition can be linked to the ability of plant growth promotion^37,38^. Similarly, we found that the soil fungal community compositions were more similar in *Trichoderma*-amended bio-organic fertilizer treatments (OF + T and SOF + T) and were significantly different from that in organic and chemical fertilizers treatments (Fig. 1e, f), which may be related to the higher yield of cabbage in these two treatments. These findings are in accordance with previous studies, which reported that different fertilization management strategies could impact crop growth and yield by regulating the indigenous microbial community diversity and composition^39,40^_._

Despite a small change in soil functioning with respect to plant growth may involve complex shifts in soil microbiome, it has also been observed that plant growth promotion may stem from changes in population densities of specific microorganisms^24,41^. Similarly, we found 88 fertilization-stimulated fungal OTUs were significantly and positively correlated with cabbage yield, of these potentially beneficial fungi, 73 and 87 fungal OTUs were significantly enriched in the *Trichoderma*-amended bio-organic fertilizer treatments of SOF + T and OF + T, respectively (Fig. 2b). Particularly, these results were further confirmed by our culture-based experiments, which found that most *Aspergillus*, *Penicillium,* and *Trichoderma* isolates belonging to potentially beneficial fungal groups could significantly promote cabbage growth. These results are in line with previous reports that some plant growth-promoting rhizobacteria (PGPR) could indirectly impact soil microbial community composition and function by stimulating the population densities of specific soil-borne microorganisms, and thereby have the potential to promote plant growth and health^24,41^. Notably, we noticed that the combined inoculation of *T*. *guizhouense* NJAU4742 together with these potentially beneficial soil-borne fungal isolates yielded a higher level of plant growth promotion effectiveness. As such, it appears that the effectiveness of *Trichoderma*-amended bio-organic fertilizer stems not only from the action of *T*. *guizhouense* NJAU4742, but also from the stimulation of fungal populations native to soil that together with *T*. *guizhouense* NJAU4742 serve to promote crop growth and yield. These findings are supported by previous reports that have shown plant growth promotion can be the product of the combined actions of diverse microbial taxa^29,42^.

Our findings suggest that *Trichoderma*-amended bio-organic fertilizer may have the potential to specifically stimulate the population densities of soil-borne beneficial fungi that contribute to plant growth. Possible explanations for these findings might the application of *Trichoderma*-amended bio-organic fertilizer introduced new metabolic capacity^43,44^, created new ecological niches^45^, induced root exudation of metabolites^46^, or competed for resources with soil native microorganisms^26^, in such a way as to stimulate or suppress particular microbial taxa, and thereby resulting in a series variation of soil microbiome and ecosystem function. Other mechanisms contributing to the observed changes in soil fungal community diversity and composition may involve the impact of chemical and organic fertilizers on the carbon/nitrogen (C/N) ratio of soil^47,48^. Previous studies have shown that organic fertilizer application can increase C/N ratio, which could be linked to the promotion of an r-selected copiotrophic life history strategy^47^ as well as an enhancement in soil microbial diversity^48,49^. By contrast, the limitation of soil carbon due to chemical fertilization pattern decrease the soil C/N ration that can be linked to a K-selected oligotrophic life history strategy^47^ and a decrease of soil microbial diversity^48,49^. Several previous studies have also reported that soil biodiversity is correlated with the maintenance of numerous ecosystem functions, and highly diverse communities may have greater plant growth promotion potential^29,50,51^. Our observation of higher fungal community diversity and crop yield in *Trichoderma*-amended bio-organic fertilizer treatments (OF + T and SOF + T) as compared to chemical fertilizer treatment (Fig. 1b, c) supports these findings.

Notably, we further observed a remarkable positive correlation between the biodiversity of soil beneficial phylotypes rather than the overall soil microbial community diversity with cabbage yield^17^, which is the correlation that we highlighted in our greenhouse experiments (Fig. 4b, c). Thus, purely increasing the diversity of the entire soil microbial community may not always stably improve crop growth and yield^52^. In natural environments, soils with more of these advantageous taxa also have greater nutrient availability, organic matter decomposition efficiency, plant productivity, and disease control abilities^16,17^. Despite the fertilization could also disrupt the favorable relationship between soil biodiversity and crop yield on cropland, these effects are always varied depending upon the type of fertilization^53^. Indeed, our findings demonstrate the importance of the diversification of beneficial phylotypes induced by bio-organic fertilization patterns in sustaining crop output.

Our study was carried out under a crop rotation system. Different crop types tend to recruit unique rhizosphere microbial communities, but this process is always limited by the composition of microbial communities in different types of soils^54^. In this case, it would be necessary and meaningful to describe in the future how to crop types mediate the *Trichoderma*-amended bio-organic fertilizer stimulating beneficial microorganisms native to soil, as well the influence of crop types on the exact mechanisms of dialog between microorganisms and how their combined action serves to confer crop promotion.

In this study, we draw the conclusion that plant development can be enhanced by *Trichoderma*-amended bio-organic fertilizers that have positive impacts on crop yield through shifting the composition of soil-borne microbiome. We noticed a favorable response by beneficial taxa, evidencing the significance of the variety of advantageous microorganisms native to soils. These findings highlight the value of the biodiversity of beneficial phylotypes in sustaining crop output, and the potential for improving crop yields through the mediation of soil-borne beneficial taxa in intensive agriculture.

## Methods

### Field site description and experimental design

The long-term field trial was performed at Nanjing Institute of Vegetable Science, in Hengxi town, Nanjing, Jiangsu Province (31°43′ N, 118°46′ E). The continuous crop rotations experiment started from September 2015, and the Chinese cabbage and wild cabbage were circularly planted. The field trial was applied in a randomized complete block design in three replicates with six treatments including (1) Control: soil amended with no fertilizer; (2) CF: soil amended with chemical fertilizer; (3) SOF: soil amended with sterilized organic fertilizer; (4) SOF + T: soil amended with sterilized organic fertilizer inoculated with *T. guizhouense* NJAU4742; (5) OF: soil amended with organic fertilizer; and (6) OF + T: soil amended with organic fertilizer inoculated with *T. guizhouense* NJAU4742 (Fig. 1a). Chemical fertilizer was mineral N (urea), P (superphosphate), K (potassium sulfate). Organic fertilizer is a composition of compounds including liquid amino acids (derived from animal carcasses) and compost (pig manure)^55^. The basic properties of organic fertilizer and sterilized organic fertilizer are as follows: organic matter 457.1 g kg^−1^, water content 28%, total nitrogen (N) 14.8 g kg^−1^, total phosphorus (P) 25.2 g kg^−1^, and total potassium (K) 20.1 g kg^−1^. The population density of strain *T. guizhouense* NJAU4742 in OF + T and SOF + T treatments was 5.0 × 10^7^ CFU g^−1^. *T. guizhouense* NJAU4742 was isolated from soil and has been widely used as a commercial biological agent in China^55,56^. Co75 γ-ray irradiation was used to sterilize organic fertilizer at Nanjing Xiyue Technology Co., Ltd., Nanjing, China. Traditional agricultural techniques were used to manage all other farm activities according to local habits. Bio-organic fertilizers (SOF + T and OF + T), organic fertilizers (SOF and OF), and chemical fertilizers (CF) with equal nutrients were applied as basic fertilizers one week before planting by using a rotary tiller in each cropping season. Detailed information about the kinds and amounts of these composts and chemical fertilizers was shown in Supplementary Table 1. Crops were watered in equal amounts according to local weather conditions^57^. Wild cabbage yield was evaluated during the full-bearing period in June 2019.

### Sample collection and DNA extraction

Soil samples were performed in June 2019 during the wild cabbage (cabbage) harvest season as significant differences of yield between different fertilization treatments were observed. Soil cores with a depth of 0–15 cm were obtained from five places to create a composite sample for the bulk soil sample. Each bulk soil sample was separately combined and filtered from a 2-mm sieve to uniformize the soil. In total, 18 bulk soil samples (6 treatments × 3 replicas) were sampled and stored at −80 °C in preparation for DNA analysis. The PowerSoil DNA Isolation Kit (Mobio Laboratories, Carlsbad, CA, USA) was used to recover and extract the total soil microbial DNA from 0.5 g of soil in accordance with the manufacturer’s instructions. Then, the quality and concentration of the DNA were measured through the spectrophotometer (NanoDrop 2000, USA).

### Real-time PCR assay and amplicon sequencing

The real-time PCR was used to measure the population density of *T. guizhouense* NJAU4742 utilizing primers (ITS1 S and ITS1 R) with the strain-specific TaqMan probe ITS TM-037 Fam: 5′-FAM-AAC TCT TTT TGT ATA CCC CCT CGC GGG T-TAMRA-3′ (FAM: 6-carboxyfluorescein, TAMRA: 6-carboxy-tetramethylrhodamine) (TaKaRa) according to Cai et al.^58^. PCR amplifications for DNA samples were performed on a 7500 Real-Time PCR System (Applied Biosystems, Pleasanton, CA, USA). Gene copy numbers were expressed as log_10_ values.

Fungal sequencing library was constructed following the procedures previously described^59,60^. The general universal fungal primers ITS1F (5′-AAC TTT YRR CAA YGG ATC WCT-3′) and ITS2 (5′- AGC CTC CGC TTA TTG ATA TGC TTA ART-3′) were used to amplify the ITS1 region from soil DNA. The library was sequenced on an Illumina Nova6000 platform and 250 bp paired-end reads were generated (Guangdong Magigene Biotechnology Co., Ltd. Guangzhou, China).

### Isolation and identification of culturable fungal isolates

Cultivable fungi were isolated from OF + T and OF soils with Rose Bengal Agar to prevent bacterial growth. Well-mixed soil weighing 20 g was added to 180 ml of sterile water (10^−1^ soil dilution), which was then serially diluted to 10^−2^, 10^−3^, and 10^−4^^61^. After that, 0.1 ml of each concentration of the soil solution was plated in media. Plates were incubated at 25 °C and checked periodically for fungal growth for up to 10 days. All fungal colonies were transferred to Potato Dextrose Agar (PDA) plates and re-incubated at 28 °C. Following replication-based purification of these fungal isolates, we used primers ITS1 (TCC GTA GGT GAA CCT GCG G)/ITS4 (TCC TCC GCT TAT TGA TAT GC) to amplify the full length of the ITS to improve the accuracy of fungal identification. These fragments were amplified and sequenced by the TSINGKE Biological Technology Company (Beijing, China), and each sequence was compared with sequences available in the NCBI GenBank database using BLAST^62^. The phylogenetic analysis of the ITS rRNA gene sequences was performed using MEGA7 and visualized with ITOL^63^ (Supplementary Fig. 1).

### Pot experiments

1. Greenhouse Experiment 1: Effect of fungal isolates on cabbage growth.
  Based on the results of fungal community analyses, twelve *Aspergillus*, nine *Trichoderma*, four *Penicillium,* and three *Fusarium* isolates were selected to examine the effect of fungal isolates on cabbage growth. Cabbage seedlings were transferred to 150-ml pots filled with sterilized substrate (vermiculite: seedling substrate: quartz sand (4:1:2)) pre-inoculated with fungal isolates *Trichoderma* sp. TRItBB, TRItED, TRItFG, TRItG, TRItGB, TRIt98, TRItA0C, t102, TRItA8mBm; *Aspergillus* sp. AStBB-A, t35, AStEF, ASt9B, AStB, AStCA, AStE8, ASt9AB, ASDF, ASE, ASDG C, ASBD; *Penicillium* sp. PENF0, 45, ENCFmBm, PENB8; *Fusarium* sp. 53, FUECmBm and FUCE, with a final inoculation density of 1 × 10^4^ spores g^−1^ substrate. A control treated with sterilized water was also established. Pots (6 replicates) were cultivated in a growing chamber with an average temperature of 25 °C, 80% relative humidity, and 16 h light/8 h dark. The plants were harvested after planting for 15 days, and the fresh weight of each plant was determined. Fungal spores were produced on Potato Dextrose Agar (PDA) plates after incubating at 28 °C for 7 days and then counted by using the blood cell counting plate method. The procedure for cabbage seeds included soaking them for 1 minute in 70% ethanol, washing them in sterile water, soaking them for 5 mins in 3% NaClO, and then washing them six times in sterile water. Before transplanting, disinfection seeds were cultivated in pots filled with sterilized substrate for 10 days.
2. Greenhouse Experiment 2: Effect of co-inoculation of *T. guizhouense* NJAU4742 plus potentially beneficial fungal isolates
  Based on the results in greenhouse experiment 1, four fungal isolates (*Trichoderma sp*. t102, *Aspergillu*s *sp*. t35, *Penicillium sp*. 45, and *Fusarium sp*. 53) with plant growth promotion capacity were selected for further study. Cabbage seedlings were transferred to 150-ml pots filled with sterilized substrate pre-inoculated with *Trichoderma sp*. t102, *Aspergillu*s *sp*. t35, *Penicillium sp*. 45, or *Fusarium sp*. 53, or a combination of *T. guizhouense* NJAU4742 with either *Trichoderma sp*. t102, *Aspergillu*s *sp*. t35, *Penicillium sp*. 45, or *Fusarium sp*. 53. The inoculation density was 1 × 10^4^ spores g^−1^ of substrate, and for two-strain treatments, the inoculation density was 0.5 × 10^4^ spores g^−1^ for each fungal isolate. Pots (six replicates) were watered with sterilized water and transferred to the growth chamber with an average temperature of 25 °C, 80% relative humidity, and 16 h light/8 h dark. The plant fresh weight was determined after planting for 15 days. The procedure for producing fungal spores and cultivating cabbage seedlings were identical to those used in the greenhouse experiment 1.
3. Greenhouse Experiment 3: Effect of microbial richness on plant growth.
  To examine the effect of microbial richness on plant growth, we performed a short-term pot experiment in which four individual fungi (*Aspergillus* sp. t35, *Trichoderma* sp. t102, *Penicillium* sp. 45, and *Fusarium* sp. 53) were set up four richness levels (1, 2, 3, and 4-strain communities altogether 15 different combinations, Supplementary Table 2). Cabbage seedlings were transferred to 150-ml pots filled with sterilized substrate pre-inoculated with these four fungi and their combinations, with a final inoculation density of 1 × 10^4^ spores g^−1^ substrate. A control treated with sterilized water was also established. Pots (six replicates) were watered with sterilized water and transferred to the growth chamber with an average temperature of 25 °C, 80% relative humidity, and 16 h light/8 h dark. The plant fresh weight was determined after planting for 15 days. The procedure for producing fungal spores and cultivating cabbage seedlings were identical to those used in the greenhouse experiment 1.
4. Greenhouse Experiment 4: Effect of microbial population density on plant growth.
  To explore the effect of microbial population density on plant growth, we conducted a short-term pot experiment in which four individual fungi (*Aspergillus* sp. t35, *Trichoderma* sp. t102, *Penicillium* sp. 45, and *Fusarium* sp. 53) were inoculated into the sterilized substrate at different densities. Cabbage seedlings were transferred to 150-ml pots filled with sterilized substrate pre-inoculated with different concentrations of *Aspergillus* sp. t35, *Trichoderma* sp. t102, *Penicillium* sp. 45, and *Fusarium* sp. 53, with final inoculation densities as follows: 1 × 10^3^, 1 × 10^4^ and 1 × 10^5^ spores g^−1^ substrate. A control treated with sterilized water was also established. Pots (six replicates) were watered with sterilized water and transferred to the growth chamber with an average temperature of 25 °C, 80% relative humidity, and 16 h light/8 h dark. The plant fresh weight was determined after planting for 15 days. The procedure for producing fungal spores and cultivating cabbage seedlings were identical to those used in the greenhouse experiment 1.

### Bioinformatic and statistical analyses

Raw sequences were handled in accordance with established procedures^55^. After removing the low-quality sequences, the forward and backward sequences for every sample were merged. The operational taxonomic unit (OTU) table was created by the USEARCH global alignment algorithm. Afterward, the remaining sequences were allocated to 4629 OTUs at a 97% similarity threshold, and chimeras were filtered. Overall, representative sequences of fungal OTUs were classified against the UNITE Fungal ITS database using the RDP classification^64^.

Statistical analysis was carried out using the R software programs (Version 4.0.5) and IBM SPSS 20.0 software program (IBM Corporation, New York, USA). Statistical tests used in this research were deemed significant at *P* < 0.05. In the field experiment, we used *t* test to determine the significant differences in yields between bio-organic fertilizer treatments (SOF + T and OF + T) and organic fertilizer treatments (SOF and OF) and applied Turkey’s test to determine the significant differences in yields and alpha diversity indexes among all treatments. The VEGAN_2.5-6 (function: diversity) package in R was used to determine alpha diversity indicators for each sample, including richness and Shannon indices. Nonmetric multidimensional scaling (NMDS) based on a Bray–Curtis dissimilarity matrix was carried out and visualized using the R vegan package to assess fungal beta diversity^65^. The analysis of variance (Adonis) was tested to evaluate the significance of different factors on community structures using the adonis function of the “vegan” package in R^66^. Multiple regression trees (MRTs) were constructed to estimate the explanatory variables responsible for differences in microbial communities^67^. The Spearman correlation (FDR < 0.05) between crop yield with the relative abundance of shared and specific OTUs was used to indicate potentially beneficial fungal groups. Network analysis was performed to explore the relationships between *T. guizhouense* NJAU4742 and potentially beneficial fungi using the “Hmisc_4.5-0” package in R and visualized in Gephi^68^. Robust correlations with Spearman’s correlation coefficients (*ρ*), >0.6 or <−0.6, and *p* values, <0.05, were performed to construct networks. Plant growth promotion by each microbial community was measured as a percent change of plant aboveground fresh weight compared to the control treatment (uninoculated microbial community). We used percentage increases in the mean squared error (MSE) to estimate the importance of these fungi or their combinations, and higher MSE% values indicate more significant variables. Random forest analysis was implemented in R. The “rfPermute_2.2” tool was used to evaluate the importance of each predictor, and the “A3_1.0.0” package was used to evaluate the significance of the models^69^. Linear regression analyses relating fungal community richness or population density to the plant biomass were performed in “basicTrendline” package^70^.

## Supplementary information

Supplementary material

## Data Availability

The raw sequence data for the ITS region of all samples were submitted to the NCBI Sequence Read Archive database (https://www.ncbi.nlm.nih.gov/) with the accession number PRJNA897574.
