# Supplementary material for: Biodiversity of the beneficial soil-borne fungi steered by Trichoderma-amended biofertilizers stimulates plant production
Source: NPJ Biofilms Microbiomes. 2023 Jul 5;9:46. doi: 10.1038/s41522-023-00416-1 (PMC10322935; doi:10.1038/s41522-023-00416-1)
Supplement: Supplementary file 1 — Supplementary material [file 41522_2023_416_MOESM1_ESM.pdf]

Supporting Information

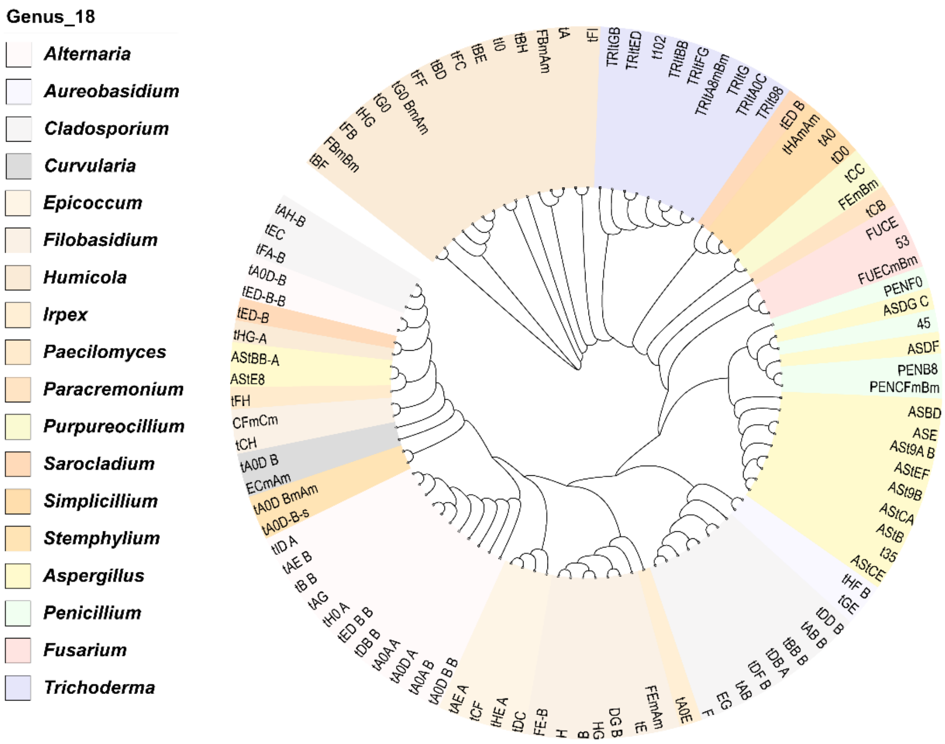

Supplementary Figure 1 Phylogenetic tree of culturable fungal isolates. Colors depict the taxonomic classification based on the genus level.

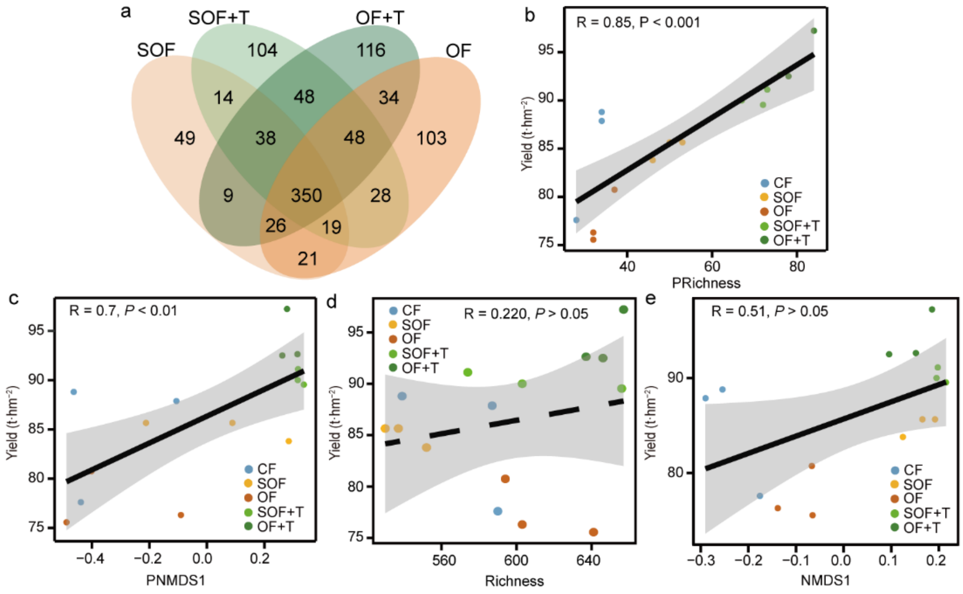

Supplementary Figure 2 Venn diagrams showing the number of fungal OTUs shared between SOF, OF, SOF+T and OF+T treatments (a). Linear regression relationship between the potentially beneficial fungal community richness index and crop yield (b). Linear regression relationship between the potentially beneficial fungal community composition (NMDS1) and crop yield (c). Linear regression relationship between the fungal community richness index and crop yield (d). Linear regression relationship between the fungal community composition (NMDS1) and crop yield (e).

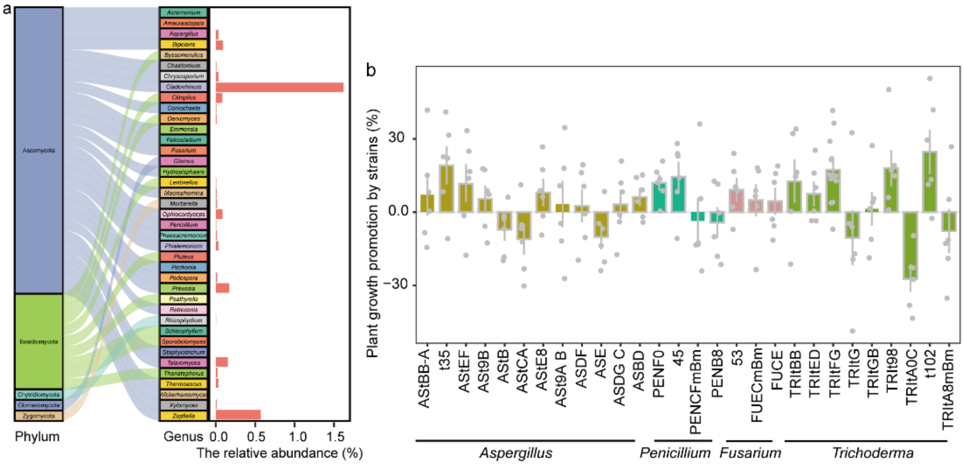

Supplementary Figure 3 The average relative abundance of potentially beneficial fungi at genus levels (a). Effects of different fungal isolates on cabbage growth (mean ± SE) (b). Gray dots represent the value of each replicate. Plant growth promotion was calculated as the percentage increase in the fresh weight of treatments compared to the control.

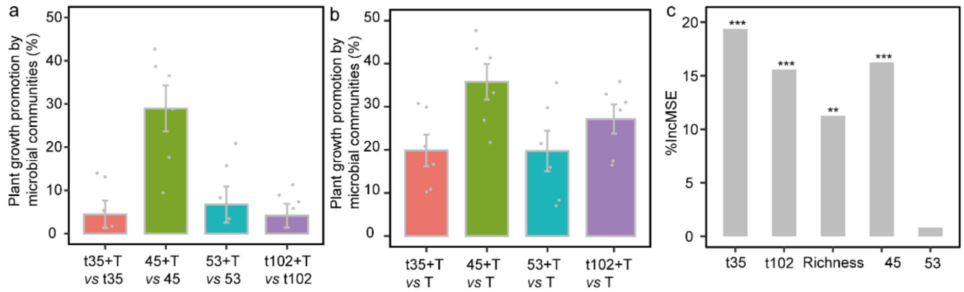

Supplementary Figure 4 Plant growth promotion of the co-inoculation of *T. guizhouense* NJAU4742 together with potentially beneficial fungal isolates when compared to potentially beneficial fungal isolates (mean ± SE) (a). Plant growth promotion of the co-inoculation of *T. guizhouense* NJAU4742 together with potentially beneficial fungal isolates when compared to *T. guizhouense* NJAU4742 (mean ± SE) (b). Gray dots represent the value of each replicate. Plant growth promotion was calculated as the percentage increase in the fresh weight of treatments compared to the control. Random forest regression showing the effects of different fungal isolates on plant biomass (c). %IncMSE indicates the percentage increase in the mean square error. Higher value of %IncMSE corresponds to a greater influence of the characteristic variable on the target. ***, $P < 0.001$; **, $P < 0.01$.

Supplementary Table 1 Fertilization scheme for different treatments

Treatments	Organic addition (pig manure) (kg/ha)	Inorganic N (Urea) (kg/ha)	Inorganic P (Calcium superphosphate) (kg/ha)	Inorganic K (Potassium sulphate) (kg/ha)
CF	0	222.0	150.0	180.0
OF	6000.0	133.2	0	59.4
OF+T	6000.0	133.2	0	59.4
SOF	6000.0	133.2	0	59.4
SOF+T	6000.0	133.2	0	59.4

Supplementary Table 2 Absence (0) and presence (1) of different microbial strains in microbial communities with varying richness levels

Group	*Aspergillus* sp. t35	*Trichoderma* sp. t102	*Penicillium* sp. 45	*Fusarium* sp. 53	Richness
1	1	0	0	0	1
2	0	1	0	0	1
3	0	0	1	0	1
4	0	0	0	1	1
5	1	1	0	0	2
6	1	0	1	0	2
7	1	0	0	1	2
8	0	1	1	0	2
9	0	1	0	1	2
10	0	0	1	1	2
11	1	1	1	0	3
12	1	1	0	1	3
13	1	0	1	1	3
14	0	1	1	1	3
15	1	1	1	1	4

Note: Each strain was present in an equal number of samples at each diversity level.

Supplementary Table 3 Effect of fungal community richness on plant growth

Richness	Microbial communities	Fresh weight (g) (M ± SD)
1	t35	0.41±0.05
1	45	0.35±0.04
1	53	0.40±0.04
1	t102	0.44±0.02
2	t35+45	0.39±0.05
2	t35+53	0.47±0.07
2	t35+t102	0.50±0.07
2	45+53	0.36±0.05
2	45+t102	0.41±0.06
2	53+t102	0.44±0.05
3	t35+45+53	0.44±0.05
3	t35+45+t102	0.46±0.02
3	t35+53+t102	0.51±0.05
3	45+53+t102	0.39±0.03
4	t35+45+53+t102	0.45±0.05

Supplementary Table 4 Effect of fungal community population density on plant growth

Population density (lgCFU/g)	Strain	Fresh weight (g) (M ± SD)
3	t35	0.43±0.05
3	45	0.37±0.04
3	53	0.36±0.06
3	t102	0.40±0.02
4	t35	0.46±0.05
4	45	0.37±0.05
4	53	0.44±0.05
4	t102	0.46±0.03
5	t35	0.47±0.03
5	45	0.51±0.05
5	53	0.37±0.04
5	t102	0.48±0.02
